# Longitudinal study of disease severity and external factors in cognitive failure after COVID-19 among Indonesian population

**DOI:** 10.1038/s41598-023-46334-2

**Published:** 2023-11-08

**Authors:** Bumi Herman, Martin Chi Sang Wong, Prawat Chantharit, Firdaus Fabrice Hannanu, Pramon Viwattanakulvanid

**Affiliations:** 1 College of Public Health Sciences, Chulalongkorn University, Bangkok, Thailand; 2 Department of Family and Preventive Medicine, Faculty of Medicine, Hasanuddin University, Makassar, Indonesia; 3 The Faculty of Medicine, JC School of Public Health, The Chinese University of Hongkong, Hong Kong, China; 4 The Faculty of Medicine, The Centre for Health Education and Health Promotion, The Chinese University of Hong Kong, Hong Kong, China; 5 School of Public Health, The Peking University, Beijing, China; 6 School of Public Health, Fudan University, Shanghai, China; 7 School of Public Health, The Chinese Academy of Medical Sciences and Peking Union Medical Colleges, Beijing, China; 8 Division of Infectious Diseases, Department of Medicine, Faculty of Medicine, Ramathibodi Hospital, Mahidol University, Bangkok, Thailand; 9 Department of Radiology, Brainstem Imaging Laboratory, Athinoula A. Martinos Center for Biomedical Imaging, Massachusetts General Hospital and Harvard Medical School, Boston, USA

**Keywords:** Infectious diseases, Neuroscience, Diseases, Health care, Neurology

## Abstract

The COVID-19 infection is assumed to induce cognitive failure. Identifying the relationship between COVID-19, the effect of vaccination and medication, and accommodating non-COVID-19 factors to cognitive failure is essential. This study was conducted in Indonesia from September 2021 to January 2023. Demographic information, clinical data, comorbidities, vaccination, and medication during COVID-19 were obtained, as well as a 6-month cognitive assessment with Cognitive Failures Questionnaire/CFQ, Fatigue Severity Score, and Generalized Anxiety Disorder (GAD-7). A Structural Equation Model explains the relationship between potential predictors and cognitive failure. The average score of CFQ after 6 months was 45.6 ± 23.1 out of 100. The severity of the disease, which was associated with vaccination status, age, previous infection, and unit of treatment (*p* < 0.05), was not related to cognitive failure (*p* = 0.519), although there is a significant direct impact of worst vaccination status to cognitive failure(*p* < 0.001). However, age, fatigue, and current anxiety were associated with higher cognitive failure (*p* < 0.001), although comorbidities and recent headaches were not significant in other models (*p* > 0.05). This study concludes that cognitive failure after COVID-19 is a multifactorial event and does not solely depend on COVID-19 severity. It is crucial to re-address the factors related to the long-term efficacy of vaccination and medication and focus on non-health factors affecting cognitive failure.

Trial Registration: NCT05060562.

## Introduction

The Coronavirus Disease 2019 (COVID-19) caused by the Severe Acute Respiratory Syndrome Coronavirus 2 (SARS-CoV-2) has become a global burden with a large number of people infected and rapid virus mutation. With 668 million patients with 6.73 million fatalities (as of January 2023)^1^, a concern regarding the long-term post-COVID-19 impact on a larger population should be addressed to prevent plummeted productivity and altered quality of life^2^. One of the main issues is cognitive failure after COVID-19.

### COVID-19 and cognitive failure

Limited evidence explains the debilitating effect of COVID-19 on the central nervous system. A proposed model in mice mimicking mild COVID-19 infection showed a neuroinflammatory condition due to microglial activation linked to the alteration of oligodendrocytes. Furthermore, the demyelination process and neurogenesis inhibition occur, particularly in the hippocampus, which could explain the disturbance of memory formation^3^. A systematic review revealed a substantial cognitive decline after COVID-19, specifically the working memory^4^. Furthermore, a 9-month follow-up of COVID-19 survivors reported that up to 26% presented with mild cognitive impairment, and 1% had a moderate alteration in cognitive function^5^.

### Vaccination and treatment on cognitive failure after COVID-19

Since the plausible mechanism of SARS-CoV-2 infection and neurodegenerative impact has been identified, there are some questions about whether vaccination and specific treatment have pivotal roles in alleviating cognitive failure through their mechanism of preventing viral replication, interaction with cell hosts and enhancing the neutralization effect. Even in the presence of the latest variant, vaccination with boosters reduces the severity^6^. In the previous study, two-dose vaccination effectively lowered selected post-COVID-19 symptoms^7^. However, the long-term effect of vaccination, booster doses, and heterologous regimens on cognitive failure in people with COVID-19 remains unanswered. It is also essential to re-evaluate the efficacy of the antiviral drug in preventing central nervous system (CNS) disorder of COVID-19 survivors, notably the best administration time.

### Individual comorbidities and socioeconomic factors on cognitive failure

Cognitive failure is a part of ageing that is affected by many factors. A study in Taiwan raised the importance of recognizing socioeconomic status in achieving good cognitive function^8^, which during the pandemic, was abruptly affected. Furthermore, chronic diseases, particularly hypertension and diabetes mellitus, are also connected to cognitive deterioration^9^. A sedentary lifestyle, obesity, and smoking also shorten the cognitive-impairment-free life expectancy^10^. It is imperative to address these factors as competing variables to explain the precise impact of COVID-19 on cognitive failure.

As the large population-based study of cognitive failure after COVID-19 is limited, this study would prove or address the possible mechanism of cognitive failure after COVID-19 by accommodating other confounders using a robust statistical model from an Indonesian cohort as the highlight and novelty of this study.

## Methodology

### Study design and setting

This longitudinal study utilized a cohort of Indonesian post-COVID-19 patients recruited since September 2021 and extracted in January 2023. Participants with a history of COVID-19 were followed up for residual symptoms. We assessed cognitive function 6 months after diagnosis and their first symptoms through an online submission system, and this platform was disseminated to the entire country through social media, health care providers, and survivor groups.

### Participants

Subjects with previous COVID-19 infection (at least thirty days from the onset or diagnosis) were diagnosed using either Indonesian Food and Drug Administration-approved antigen test for the anterior nasal sample (sensitivity over 80%)^11^ or real-time polymerase chain reaction (RT-PCR) of the nasopharyngeal, nasal, or pooled samples^12^. These procedures were performed by trained staff, as the self-administered antigen test was not recognized during the study period. This study covered all types of patients, from asymptomatic to hospitalized. After the provision of consent given by the participants, a follow-up cognitive assessment was made, and those without responses of expected outcomes were omitted. Aside from having self-reported mental illness and stroke, there is no other exclusion or limit for those with specific morbidities, age, and digital literacy. Moreover, the snowball sampling approach recruited more individuals to this cohort.

### Variables and measurement tools

Demographic characteristics, health behaviour, chronic diseases, and other comorbidities were the baseline information covered in the questionnaire. Additionally, this study recorded the latest COVID-19 episode, including date and diagnostic methods, duration of symptoms, type of medication, vaccination status, type of vaccine, and time between doses. Investigators also identified the date from vaccination to infection and the type of care received (home isolation, hospitalization, or both). These variables may interact with each other and affect the relationship between COVID-19 and cognitive failure.

This study cited the definition of cognitive failure as a cognitive mistake made while performing a task that a person would typically complete successfully in daily life. Problems with memory, perception, and focus are signs of cognitive failure^13^.

This study measured the cognitive failure 6 months after the COVID-19 infection. Investigators implemented the assessment using a questionnaire that is in proximity to clinical cognitive assessment, the Cognitive Failures Questionnaire (CFQ)(14), consisting of 25 5-Likert scale questions. The responses range from 0 to 4, where a higher number indicates frequent cognitive failure events. The Indonesian version of this questionnaire was derived from a study with a Cronbach alpha value of 0.942^14^. Furthermore, one study disclosed three domains in the questionnaire which are forgetfulness (related to something known or planned), distractibility (alteration of attention and focus), and false triggering^15^.

Investigators assessed other residual COVID-19 symptoms and neuropsychiatry conditions to identify the possible contribution of these symptoms to cognitive failure. Investigators evaluated any presence of residual symptoms after the first onset or diagnosis using a set of measurement tools such as the Fatigue Severity Scale (FSS)^16^ over the past 6 months, Generalised-Anxiety Disorder (GAD-7) to screen for recent anxiety and a 10-Likert scale question to identify its incidence over the past two weeks.

### Bias

Investigators admit that the quality of baseline data was heavily affected by recall bias. Therefore, participants were required to provide the answers based on the observation chart, written source (such as medical records summary), and through the PeduliLindungi application (for data related to testing, vaccinations, and individual information).

Participants might experience reinfection. Hence, only the last episode of COVID-19 should be provided as the response. By applying this approach, the investigators could not examine in detail the previous infection and, possibly, its latent impact on cognitive function.

Investigators knew that robust cognitive assessments such as Cambridge Neuropsychological Test Automated Battery (CANTAB) could provide objective cognitive results. However, the investigators considered the survey exhaustion and other factors affecting the response, such as internet connection and adaptation to the measurement tools. The questionnaire was deemed sufficient to represent the domains tested by these standardized clinical tests.

### Study size

There is limited information on long-term cognitive situations after COVID-19. However, a study of CFQ application to neurosarcoidosis (a subset of sarcoidosis, a multi-inflammatory systemic disorder) shows a mean score of 45.6 ± 20.7^13^. Therefore, investigators assumed that the variance of cognitive failure values of COVID-19 survivors is approximately 20.7. Using the equivalence formula from the assumed score, with 5% type 1 error, 90% power of the study, the absolute mean difference between the assumpted mean and sample means of 1, a 5% equivalence limit, and 10% listwise deletion of incomplete response, a total of 5653 participants should be drawn from the cohort.

### Quantification and discretization

Several data were presented as discrete variables, including the duration of symptoms and administration of drugs. Moreover, to assume the possible variant, the investigators matched the date of diagnosis with the variant surveillance reports issued by the Ministry of Health according to surveillance week and region. Other continuous data were kept at their original values. Each type of vaccine possesses different efficacy^17^. Hence an ordinal level of this variable was made, where a higher ordinal level of vaccination means worse vaccination status (unvaccinated) (Supplementary Data 1).

### Statistical method

**Figure 1:**
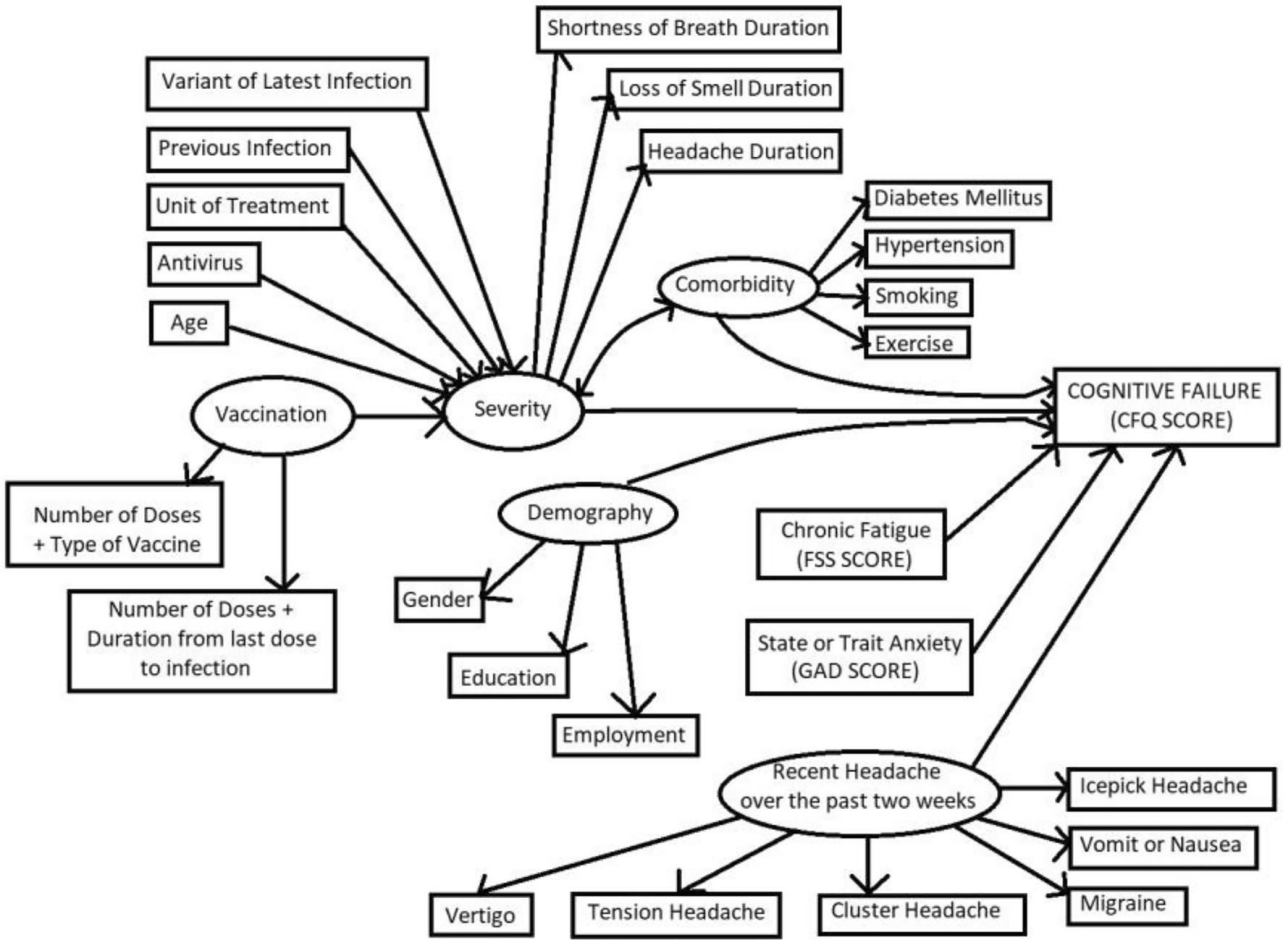
Proposed Model of Cognitive Failure post-COVID-19. This model comprises four exogenous latent variables (circle variables), and one variable acts as both an exogenous and endogenous latent variable (severity). The final endogenous factor is cognitive failure. Factor loadings of the latent variable are presented with diverging arrows from the latent variable (Recent Headache with six elements. Demography with three factors. Vaccination with two factors. Severity with three factors, and comorbidities with four factors). Converging arrows indicate the impact of the exogenous variable on the endogenous factor.

The analysis involved participants with complete responses; hence no imputation and other missing data analysis were undertaken. Descriptive statistics and bivariate analysis of variables were conducted before the final analysis. We performed the structural equation model. First, the whole model was built (Fig. 1) and simplified by the trimming approach. The structure of the model consists of exogenous variables, latent variables, and CFQ score as endogenous factors. The assumption was that vaccination, medication, virus variant, and comorbidity might affect the severity of COVID-19 and, eventually, cognitive failure. Aside from COVID-19, demography, chronic fatigue over the past 6 months, recent headaches, and anxiety may affect the cognitive situation. The investigators constructed latent variables with confirmatory factor analysis. The selection of the explanatory factors was based on bivariate analysis and model fitting assessed using the Comparative Fit Index (CFI), Tucker-Lewis Index (TLI), Standardized Root Mean Square Residual (SRMR), and Root Square Mean Error of Approximation (RMSEA). The Lavaan library in R performed the analysis and produced the final plot.

### Ethical approval

This study was authorized by the Hasanuddin University Research Ethics Review Committee for Research Involving Human Research Participants (full-board review number 758/UN4.6.4.5.31/PP36/2021). We confirm that all procedures, particularly the clinical data collection, were performed following relevant guidelines and regulations. Informed consent was obtained from all participants when they provided information to the cohort. We appropriately de-identified, stored, and used the data while respecting confidentiality. Any circumstances that needed immediate action were directed to a professional. This research is a subset of a clinical trial with the identifier NCT05060562 on clinicaltrials.gov.

## Results

**Figure 2:**
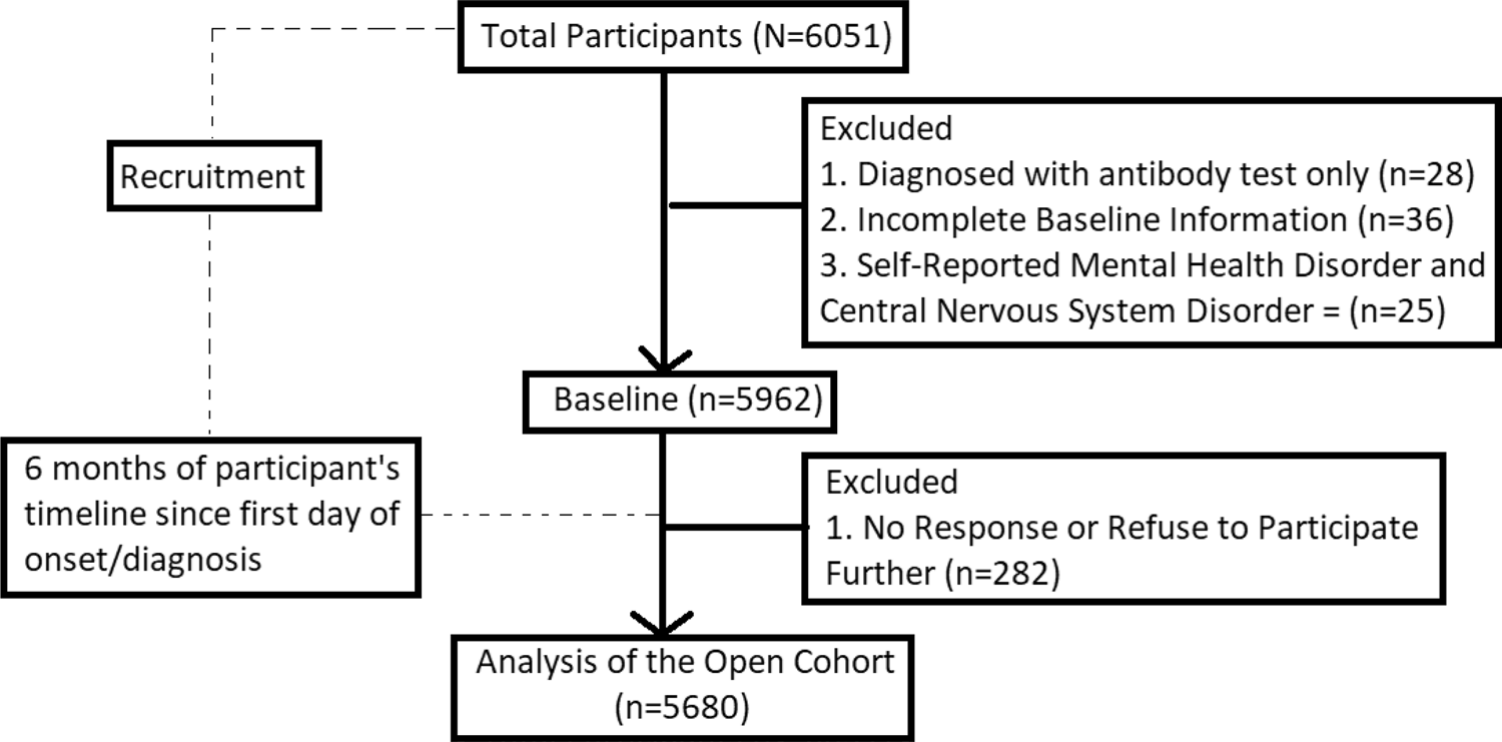
Timeline of the Dynamic Cohort. From the total participants recruited until January 2023, exclusion was made following the eligibility of the study as well as the lost-to-follow-up. Note that the 6-months timeline is the individual timeline, not the study timeline.

A total of 5680 participants were involved in this study. The average age was 28.36 ± 8.96 years (18–65 years old). This cohort was dominated by participants aged less than 30 years old (69.9%), followed by 30–40 years old (18.7%), 40–50 years old (8.3%), and above 50 years old for 3.1%. The distribution of sex assigned at birth was almost equal (49.9% male). As 30.2% of participants were undergraduates and graduates, and 5.3% of the participants were medical staff currently working at healthcare facilities. The distribution of cases follows the population density of each island, where participants from Java dominated the cohort (34.5%), followed by Sumatera (25.5%), Kalimantan (12.8%), Sulawesi (12.5%), Maluku and Papua (8.1%), and last with Bali and Nusa Tenggara (6.6%). Real-time PCR detected 41.1% of cases, followed by antigen test (40.5%) and RT-PCR + Antigen test (18.4%). Figure 2 depicts the selection of participants from the primary cohort.

The mean CFQ score in this cohort was 45.6 ± 23.1. Table 1 describes the distribution of variables according to the mean CFQ score. The selection of these potential predictors was based on the plausible mechanism of these factors to cognitive failure.

**Table 1:** Baseline characteristics of participants.

Variable	Subset	CFQ below mean	CFQ above mean	*p*-Value	*p*-Value raw CFQ
Age	Mean ± Standard Deviation	28.89 ± 8.91	28.06 ± 8.70	< 0.001	< 0.001
Body mass index	Mean ± Standard Deviation	23.41 ± 3.29	23.41 ± 3.42	0.844	0.154
Gender	Male	1439 (50.8)	1396 (49.2)	0.426	0.111
	Female	1414 (49.7)	1431 (50.3)		
Education	Up to Junior High School	184 (47.2)	206 (52.8)	0.036	0.044
	Senior High School	1096 (49.1)	1138 (50.9)		
	College/Diploma	717 (53.5)	623 (46.5)		
	University Graduates	856 (49.9)	860 (50.1)		
Employment	Unemployed	551 (48.7)	580 (51.3)	0.006	0.024
	Freelancer	894 (48.0)	968 (52.0)		
	Workers with fixed working hours	1121 (51.5)	1054 (48.5)		
	Medical staff working in a non-medical setting	125 (59.2)	79 (40.8)		
	Medical staff working in a medical setting	162 (53.8)	139 (46.2)		
Exercise	< 1 time per week	1980 (49.9)	1986 (50.1)	0.237	0.092
	1–3 times per week	755 (51.7)	705 (48.3)		
	> 3 times per week	118 (46.5)	136 (53.5)		
Smoking	Never	1908 (49.3)	1961 (50.7)	0.131	0.028
	Former Smoker	342 (52.4)	311 (47.6)		
	Current Smoker	603 (52.1)	555 (47.9)		
Diabetes mellitus	No	2615 (50.6)	2551 (49.4)	0.173	0.380
	Controlled by medication	112 (46.7)	128 (53.3)		
	uncontrolled	126 (46.0)	148 (54.0)		
Hypertension	No	2359 (50.9)	2275 (49.1)	0.075	0.473
	Controlled by medication	182 (48.8)	191 (51.2)		
	uncontrolled	312 (46.4)	361 (53.6)		
Antiviral agent	< 24 h after diagnosis	2304 (50.8)	2227 (49.2)	0.250	0.002
	24–72 h after diagnosis	144 (48.8)	151 (51.2)		
	> 72 h after diagnosis	346 (46.9)	391 (53.1)		
	Not received	59 (50.4)	58 (49.6)		
Unit of treatment	Self-Isolation	2545(49.9)	2555 (50.1)	0.044	0.056
	Isolation + Hospital Referral	274 (51.8)	255 (48.2)		
	Full Hospitalization	34 (66.7)	17 (33.3)		
Type of vaccination before the infection, regardless of the number of doses	Unvaccinated	1048 (42.2)	1437 (57.8)	< 0.001	< 0.001
	Inactivated Vaccine	902 (55.7)	718 (44.3)		
	Viral Vector	272 (53.0)	241 (47.0)		
	mRNA	423 (58.7)	298 (41.3)		
	Heterologous	208 (61.0)	133 (39.0)		
Last vaccine dose to infection regardless of the number of doses	1–30 days after the last vaccination	251 (53.9)	215 (46.1)	< 0.001	< 0.001
	31–60 days after the last vaccination	262 (56.6)	201(43.4)		
	61–90 days after the last vaccination	318 (58.1)	229 (41.9)		
	91–120 days after the last vaccination	195 (55.7)	155 (44.3)		
	121–180 days after the last vaccination	241 (56.6)	185 (43.4)		
	> 180 days after the last vaccination	538 (57.1)	405 (42.9)		
	Unvaccinated	1048 (42.2)	1437 (57.8)		
Occurrence of infection	Not received / before the first dose	1048 (42.2)	1437 (57.8)	0.167	< 0.001
	after the first dose	384 (55.3)	311 (44.7)		
	after the second dose	1168 (56.6)	897 (43.4)		
	after the third dose	228 (56.6)	175 (43.4)		
	after the fourth dose	25 (78.1)	7 (21.9)		
Previous infection wave	No	1944 (51.7)	1818 (48.3)	0.001	< 0.001
	Wild type	541 (47.3)	602 (52.7)		
	Alpha and Beta	101 (51.5)	95 (48.5)		
	Delta	173 (50.7)	168 (49.3)		
	Omicron	94 (39.5)	144 (60.5)		
Latest variant	Wild type	500 (41.3)	712 (58.7)	< 0.001	< 0.001
	Alpha and Beta	194 (54.0)	165 (46.0)		
	Delta	1007 (50.0)	1009 (50.0)		
	Omicron	1152 (55.0)	941 (45.0)		
Fatigue severity score	Mean ± Standard Deviation	23.53 ± 12.36	26.23 ± 10.87	< 0.001	< 0.001
Generalized anxiety disorder	Mean ± Standard Deviation	5.15 ± 3.96	7.05 ± 4.28	< 0.001	< 0.001

Categorical data were tested using the chi-square test, except continuous data (Mann–Whitney test). The significant level at *p*-value < 0.05. the *p*-value of CFQ raw score as a dependent variable was tested using the Kruskal Wallis test and Spearman test.

From Table 1, study participants with higher cognitive failure were significantly younger and graduated from junior high school or lower, compared to higher education levels (*p* < 0.05). Medical workers were less likely to have higher cognitive failure. There were no significant differences in exercise, smoking, and prescription of any antiviral agent, but there was an insignificant risk of uncontrolled hypertension, diabetes, and higher cognitive failure (*p* > 0.05). Those with a history of reinfection, particularly with newer variants and the latest infection caused by earlier variants, were more likely to have a higher cognitive failure (*p* < 0.05). Regarding vaccination, unvaccinated people were prone to severe cognitive outcomes (*p* < 0.05). Interestingly, a proportion of higher cognitive failure was seen in people who underwent self-isolation (*p* < 0.05). People with a higher level of fatigue within 6 months and higher anxiety over the past two weeks tended to have a higher cognitive failure (*p* < 0.05).

However, by treating the CFQ as a raw score (assuming that mean CFQ score discriminant ability is unknown), smoking was associated with a higher score of CFQ (*p* = 0.024). Moreover, antiviral prescription time was also associated with CFQ score (*p* = 0.002) as well as the occurrence of the infection, but not with the unit of treatment (*p* = 0.056). Nevertheless, the investigators included all variables in the initial SEM model and the trimming approach was applied.

**Figure 3:**
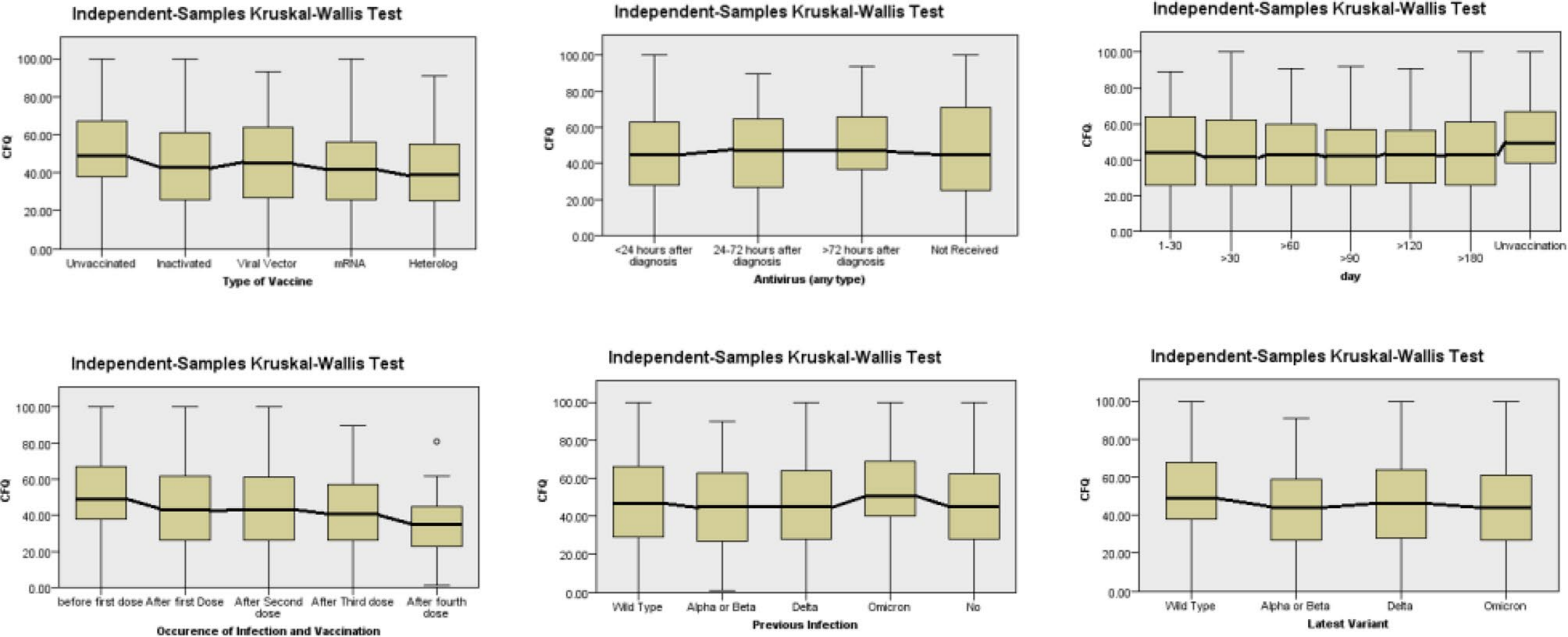
Comparison of CFQ Score in each variable. Six separate figures illustrate the comparison of CFQ score in the subset of selected variables, including the type of vaccination received before infection, time of antivirus prescription (but not time of initial ingestion), days of last dose injection to infection, the occurrence of infection according to last vaccination doses, previous infection, and latest period of infection.

Figure 3 depicts the CFQ score of some variables with a *p*-value below < 0.01. Those people who remain unvaccinated and infected, those with a history of omicron infection, or those infected with wild-type variants had a higher CFQ score. Despite having a significant *p*-value, the median CFQ score between four different antivirus prescription times shows a similar trend.

The investigators proposed a model using linear structural relation (LISREL), as shown in Fig. 1. A cutoff of a *p*-value of more than 0.2 and plausibility was applied for variable selection; hence, re-specification of the model was performed. There are four latent exogenous variables in the model. The first latent variable is recent headache, which consists of five different types of headache and one associated symptom, namely tension, cluster, migraine, vertigo, and ice pick headache followed by vomit and nausea (Fitness index CFI 0.966, TLI 0.944, SRMR 0.025) Second is comorbidities, which consists of exercise, smoking, diabetes mellitus and hypertension (CFI 0.992, TLI 0.976, SRMR 0.018). This paper wants to address the effect of vaccination. Hence, the type of vaccination, days to infection, and occurrence of infection were merged into two variables (dose-day and dose-type) and created vaccination as a latent exogenous factor. The direction of the vaccination variable was the longer duration between doses and the lower number of doses received was defined as worse vaccination status. Specific symptoms (Shortness of breath, loss of smell, and headache) construct the severity, a latent exogenous factor (CFI 1.0, TLI 1.0, SRMR < 0.001). Since two demographic factors were significantly associated with CFQ, these two variables represent a latent demography variable. Cognitive failure is assumed to be affected by severity, demography, fatigue condition, anxiety level, and recent headache, while severity is predicted by age, previous infection, latest variant, and treatment unit.

**Figure 4:**
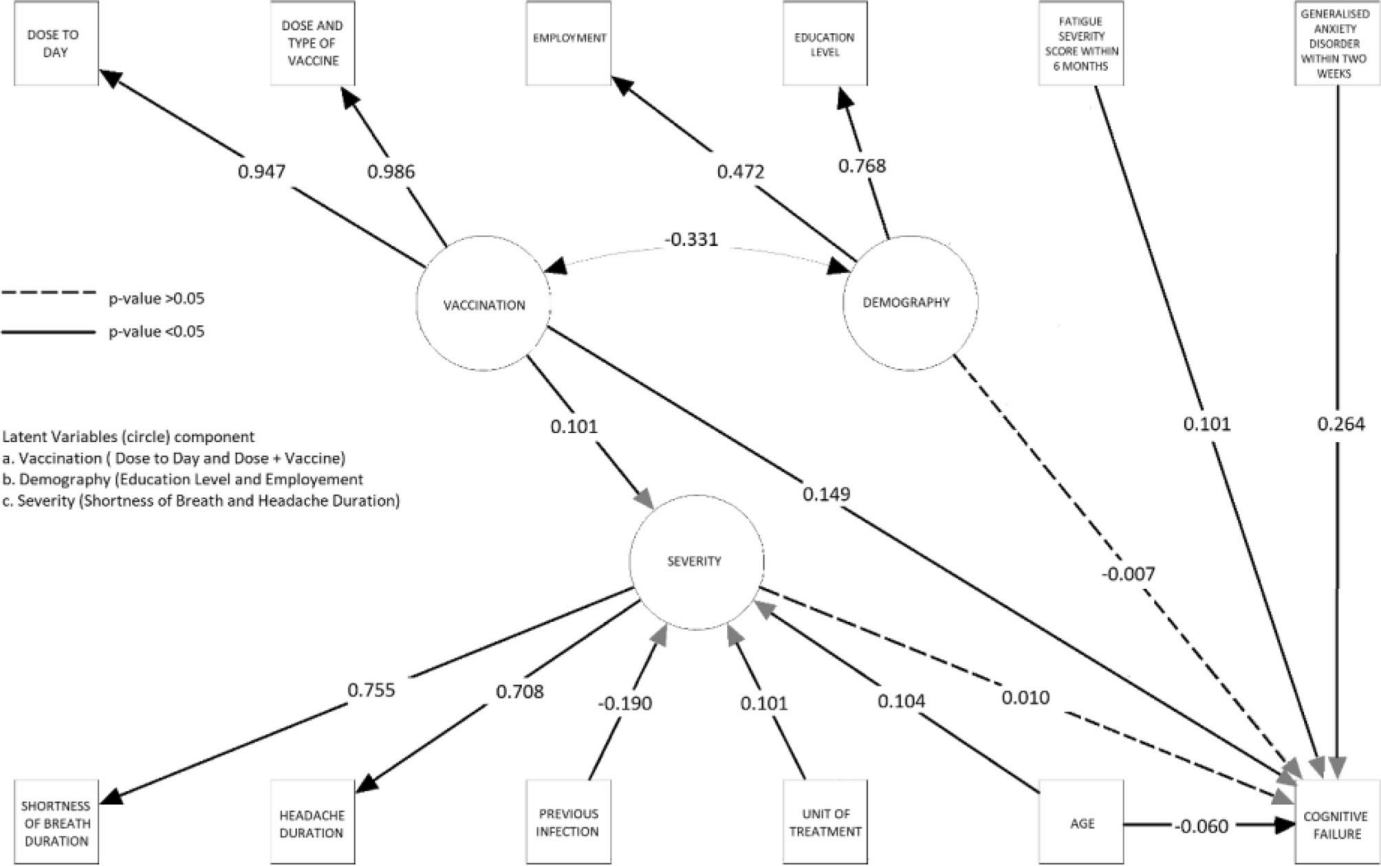
The Linear Structural Relation Of The Final Model. Variables in the circle are the latent variables constructed by other variables (as written in the figure). The line shows the *p*-value of the relationship where the interrupted line indicates a non-significant relationship and the intact lines were significant. The number represents the standardized estimate of the parameters (β), except for the circular bi-directional arrows, which represent the variance of latent variables. Bidirectional arrows present the covariance between demography and vaccination.

There are three proposed models with good fitness indices. However, one model possessed an acceptable fitness index (CFI 0.958, TLI 0.940, SRMR 0.044, and RMSEA score of 0.056, although χ^2^/df = 18.8). Figure 4 depicts the LISREL of the model.

Table 2 elaborates on the estimates of each variable of the final model. Regarding the severity of the disease, represented by the duration of shortness of breath and headache during the latest COVID-19 episode, unvaccinated people were at risk of having severe disease compared to those receiving more doses and a heterologous regimen. Furthermore, there was a link between severe illness and hospitalization, older age, and absence of previous infection or previous infection with earlier variants.

**Table 2:** Parameter estimates of the final model.

Structure	Variables	Estimate	Standard error of estimate	z-Value	*p*-value	Standardized estimate (β)	R-Square
Latent variables							
Severity	Shortness of Breath duration	1				0.755	0.570
	Headache duration	0.869	0.06	14.563	< 0.001	0.708	0.501
Vaccination	Doses + Day to Infection	1				0.947	0.897
	Doses + Type of Vaccine	0.636	0.010	62.536	< 0.001	0.986	0.973
Demography	Employment	1				0.472	0.223
	Education	1.532	0.127	12.071	< 0.001	0.768	0.590
Regression							
Severity	No previous infection	− 0.076	0.007	− 11.467	< 0.001	− 0.190	0.066
	Increase of Age	0.008	0.001	6.481	< 0.001	0.104	
	Hospitalization	0.194	0.031	6.337	< 0.001	0.101	
	Unvaccinated	0.014	0.002	6.308	< 0.001	0.101	
CFQ score	More Severe Disease	0.338	0.525	0.644	0.519	0.010	0.147
	FSS score	0.199	0.031	6.363	< 0.001	0.101	
	GAD Score	1.450	0.086	16.822	< 0.001	0.264	
	Highly educated and working in a medical setting (Demography)	− 0.336	0.807	0.227	0.677	− 0.007	
	Worse Vaccination Status	0.713	0.065	10.895	< 0.001	0.149	
	Age	− 0.158	0.033	− 4.781	< 0.001	− 0.060	
Covariance	Worse Vaccination Status vs. Demography	− 0.771	0.068	− 11.389	< 0.001	− 0.331	
Indirect effect	Type of Treatment to CFQ Via Severity	0.005	0.007	0.641	0.521	0.001	
	Worse Vaccination Status to CFQ Via Severity	0.065	0.102	0.641	0.521	0.001	

There were four significant predictors of cognitive failure. It is interesting to see that younger age was associated with higher CFQ score (*p* < 0.001). Cognitive failure was linear to higher chronic fatigue and anxiety levels but not associated with demographic features (*p* = 0.677). Further covariance analysis showed that people with lower education and unemployed tended to have worse vaccination status (as represented by covariance analysis *p* < 0.05). In the previous model, higher comorbidities were independent of severity and cognitive failure (Supplementary Table S1). Recent headaches, which could represent any CNS disorder, were also not associated with cognitive failure in the full model (Supplementary Table S1), which was then omitted from the final model. A significant relationship was also seen between the latest variant and severity (*p* = 0.046 Supplementary Table S2) on the non-fit model, where the newest variant was associated with a shorter duration of each symptom (Supplementary Table S3), which shows possible collinearity. To produce a better model, the variable latest variant was omitted from the final model as its *p*-value is the highest among other predictors of severity (Supplementary Table S4).

Finally, in the Structural Equation Model, there are several factors linked to cognitive failure in the post-COVID-19 period. The severity of the disease as the latent variable was not associated with the CFQ score 6 months after infection (*p* = 0.519) as well as education and working position (*p* = 0.677). However, worse vaccination status as a latent variable was associated with higher CFQ scores (standardized estimate β = 0.149 *p* < 0.001). External factors such as age (standardized estimate β = − 0.060 *p* < 0.001), anxiety (GAD score, standardized estimate β = 0.264 *p* < 0.001) and fatigue (FSS score, standardized estimate β = 0.101 *p* < 0.001) also affect the CFQ score). Among the four factors, according to the standardized estimate β, generalized anxiety is the most influential factor of cognitive dysfunction. However, these five factors only explain 14.7% of the variance of the CFQ score. Further analysis according to each domain of CFQ score revealed similar significant factors including forgetfulness (Supplementary Table S3), distraction (Supplementary Table S4), and false trigger (Supplementary Table S5). However, fatigue score was not associated with distraction (*p* = 0.051).

## Discussion

The average score and median score of CFQ was 45 out of 100, which explains that cognitive failure after 6 months should not be underestimated. This cohort was dominated by younger age (< 30 years) as this group is more vulnerable and transmitting infection^18^ due to higher mobility and is among the non-priority groups for booster vaccination. This study revealed that younger people were prone to cognitive failure. Younger ages are sensitive to changes in cognitive function as they are heavily affected by stress, anxiety, and depression when responding to their surroundings^19^. This could be an underlying issue that CFQ consists of questions related to daily activities that most elder people would not do, and if they do, it would not be as complex as what the younger are engaged in. Hence younger people will be more sensitive to smaller changes of limitation in doing the reported activities in CFQ and thus, show a higher fluctuation of CFQ score.

The consideration to select the three symptoms (loss of smell, shortness of breath, and headache) to build a latent variable representing COVID severity was based on the plausibility of SARS-CoV-2 infection to central nervous system (CNS) perturbation, the possible ascending transmission through olfactory nerve^20^ which represent by loss of smell, shortness of breath, which could lead to hypoxia, and the possible CNS inflammatory condition, roughly marked by headache^21^. In the final model, loss of smell was omitted due to insignificance in predicting CFQ class (*p* > 0.05), provided in Supplementary Table S6. In theory, specific variants are linked to severe disease, and a significant relationship was seen between the latest variant and severity (Supplementary Table S6) where people infected during the delta period had a bigger proportion of having a longer duration of symptoms, compared to other variants. However, including the newest variant variable in the structural model did not produce a fit model as the goodness of fit indices is below the recommended value (Supplementary Table S2) and improvement was seen when omitting the latest variant (Supplementary Table S7). A better model without loss of smell and the latest variant is shown in Supplementary Table S8, showing a more acceptable cognitive failure model.

Severity was assumed to be the mediator of other variables to cognitive failure—the absence of previous infection or previous infection with newer variants negatively correlated with severity. A study revealed frequent reinfection with the latest variant, although the severity remains lower than the first infection^22^. This study also identified the benefit of vaccination in reducing the severity, particularly for those who received booster doses with a heterologous regimen and had a shorter duration from vaccination to infection, which is in line with a meta-analysis^23^. It is important to note that the association between severity and hospitalization should be interpreted cautiously, as severe diseases are likely to be admitted. In bivariate analysis, the prescription and administration of antiviral agents were maximized in hospitalized individuals (*p* < 0.05) (Supplementary Table S9). In practice, vaccinated people with mild symptoms are less likely to receive antiviral agents than those with moderate and severe diseases. Table 1 indicates that mild cases who underwent self-isolation experienced higher cognitive failure, which ignites the role of antiviral agent prescription to limit viral ascending to CNS and preventing cognitive impairment. Nevertheless, there was no significant association between disease severity and cognitive failure. Receiving vaccination or antiviral agent is not enough as it should be done and administered in an appropriate time.

Early and rigorous viral clearance is essential to reduce the severity and subsequent CNS injury, affecting cognitive function through rapid viral neutralization with antibodies and suppression of viral replication with antiviral agents. A study in Japan demonstrated the reduction of median neutralizing antibody (NAb) titer antibody up to 44.3%, 12 months after wild-type infection and undetected at 35.5 months^24^. Furthermore, the number of Nab against ancestral and delta variants in vaccinated individuals was 7.8 and 4.0 higher than in unvaccinated individuals. This study also revealed a faster time to reach peak concentration among those vaccinated participants (two weeks versus three weeks). However, the number of NAb titers was not associated with the duration of viral clearance (*p* < 0.267). It should be noted that the participants were vaccinated with inactivated virus vaccine and protein subunit vaccine^25^. With the mRNA vaccine, inhibition against live omicron-type viruses was the lowest among other variants and remained moderate using the serum of patients who received a third dose and took it after three weeks^26^. A heterologous regimen (inactivated + booster mRNA) demonstrated better protection against omicron by enhancing the memory B cell and specific T cell response compared to the homologous inactivated vaccine^27^. The finding concludes an insignificant indirect vaccination effect on cognitive failure by reducing disease severity (*p*-value 0.521 in Table 2). However, when considering a direct effect on CFQ, worse vaccination status as a latent variable was associated with higher CFQ scores (standardized estimate β = 0.149 *p* < 0.001) In short, only relying on neutralizing antibodies attained by vaccination is insufficient against the virus and severity prevention, but not in cognitive dysfunction prevention.

It is crucial to address how fast the dissemination of the virus to multiple organs versus the viral clearance is. The heatmap of SARS-CoV-2 RNA showed that the distribution of subgenomic RNA copies during the first four days of illness was higher in the dura mater and cerebral cortex. Furthermore, from 5 to day 13, at least 0.05- < 0.1 copies per nanogram of RNA SARS-CoV-2 input was found in the basal ganglia and even higher in the cerebral cortex^28^. Considering the slower duration to reach the peak level of neutralizing activity of antibodies achieved by vaccination, relying on the vaccination-attained antibody, particularly in high-risk individuals, is insufficient. It is relevant to consider the early initiation of antiviral agents to boost viral clearance. However, a systematic review of molnupiravir versus placebos showed no significant difference in mean viral load and duration of viral clearance (14–15 days) in unvaccinated individuals and people with mild-moderate non-hospitalized cases^29^. Another systematic review of favipiravir exhibited faster viral clearance for up to 7 days after initiation^30^, although a study on mild COVID-19 did not show a better duration of viral clearance^31^. In this cohort, various antiviral agent was prescribed, but the time from diagnosis to prescription and ingestion was unidentifiable. A study emphasized that a golden period of treatment from onset to hospitalization is within four days^32^ to reduce the severity, which could explain the detrimental efficacy of antiviral agents. Moreover, this study revealed a significant connection between increasing age and the severity as older age was linked to higher viral load^33^ and severe inflammatory conditions^34^. Thus, considering these factors, it is cogitable why the indirect effect of the unit of treatment on cognitive failure via severity of disease was not significant (*p*-value 0.521 in Table 2).

This study also identified other factors that were connected to cognitive failure. Fatigue over the past 6 months was related to cognitive failure. The FSS questionnaire also assessed the fatigue impact on the duties and responsibilities linked to cognitive function. Fatigue was one of the most common post-COVID-19 symptoms, and several theories postulated the origin of fatigue and cognitive dysfunction through indirect mechanisms, mainly the inflammatory condition and altered metabolism of neuron cells^35^. Furthermore, fatigue is hypothetically affected by conditional and physiological aspects related to mental capacity, task complexity, and surrounding conditions^36^. Following the theory mentioned above, the GAD, which reflects state anxiety and possibly trait anxiety, could be a competing factor that affects cognitive function, particularly overstimulating alerting and orientation or alteration in the executive control network^37^, and it is proven to affect cognitive failure in this study. It is pivotal to assess people's psychosocial condition during the pandemic comprehensively.

This study constructed a latent demography variable as mental capacity and decision-making are linked to education level and organizational factors. A review shows that highly educated people seem to have better cognitive functions^38^ but are mostly affiliated with higher-pressure occupations. Long working hours were associated with negative cognitive performance, particularly in middle age^39^. A study comparing the psychological distress about COVID-19 between health workers and the general population showed an alarming rate of stress and anxiety in health workers^40^. However, the final model showed a non-significant relationship between demography and cognitive failure (*p* = 0.677), which could imply variety in working conditions and individual coping mechanisms.

There is a question of whether a recent headache is sequelae of COVID-19. This study identified the headache that occurred before the cognitive assessment to eliminate possible CNS disorder that affects cognitive function. A study pointed out that at least 20% of COVID-19 patients will develop chronic headaches for up to 9 months^41^. Full vaccination status could reduce chronic headaches after 90 Days from COVID-19 first onset^7^, which could illustrate the effect of vaccination on cognitive failure via the prevention of chronic headaches. However, the additional model shows an insignificant indirect impact of vaccination on cognitive failure via prevention of recent headaches (*p*-value 0.217 in Supplementary Table S7). To summarize, cognitive failure after COVID-19 is not solely a subsequent effect of disease severity but is highly affected by other external factors.

Some limitations should be addressed despite the study covering longer periods of observation, more representative participants, and various vaccination statuses. It is vital to compare cognitive failure among people who never get infected. Due to the higher number of participants and limited resources, this study could not conduct such a test. Although this study excluded people with self-reported mental illness and CNS disturbances such as stroke, there is still a possibility of undetected CNS disorders in participants that should be clinically ruled out. Even with the additional questions to identify the headache that might be related to CNS disease, this procedure was less appropriate to justify the actual clinical situation. Furthermore, to see the true effectiveness of antiviral agents in reducing the severity and cognitive failure, the duration of symptoms should be measured starting from the time of receiving medication, not recording the entire duration of symptoms.

There is a question of whether this questionnaire is suitable for measuring cognitive failure in COVID-19. A study implemented cognitive assessment using Perceived Memory and Attentional Failures Questionnaires (PerMAFaq) during the COVID-19 period but focused on the mental health of healthy people^42^. Moreover, one study evaluating the effect of COVID-19 on cognitive failure at work applied three measurement tools^43^ which are not linear with this study's objectives and settings. This study is one of the initial studies that utilized the CFQ to assess post-COVID-19 symptoms. Although using proxy tools for a more reliable clinical assessment of cognitive function, there is a lack of clinical correlation between CFQ and CANTAB^44^. To justify the use of CFQ, the authors explored the connection between the changes in brain anatomy and physiology that are common after COVID-19 and the cognitive failure measured by CFQ. Disturbance in the Gamma-Aminobutyric Acid (GABA)ergic system occurs in COVID-19, marked a depletion of GABA and dysregulation of the GABAergic system^45^**.** Moreover, increased grey matter volume was common among people with previous COVID-19 infection^46^. A study to explore GABA and grey matter volume (GMV) in connection to cognitive failure using CFQ showed that there wass a negative correlation between GABA and CFQ, as well as positive correlation of GMV and CFQ^47^. Thus CFQ is appropriate in measuring the cognitive failure after COVID-19.

## Conclusion

This study demonstrates the structural relationship of factors associated with cognitive failure among COVID-19 survivors and this study indicates that cognitive failure after COVID-19 is not solely because of COVID-19, but rather a combination of external, psychological factors and 85.3% of unexplored factors. Vaccination remains essential to reduce the severity, although doses, type, and timing of administration should be maximized to enhance the long-term benefit of cognitive dysfunction prevention. It is also recommended to assess the non-health circumstances that influence the mental health of individuals (including fatigue and anxiety), as during the COVID-19 pandemic, people encounter financial hardship and are exposed to psychosocial stressors. To address some unexplored variance (85.3%), further suggestions should be considered, including measuring the anatomical changes of the brain that is prevalent among COVID-19 survivors, (this include the assessment of cognitive-related area and GABA function), applying repeated measures of external factors, including job stress and quality of life and conducting objective measurement of chronic disease status and progression, including complication and medication which may affect the cognitive failure.

### Supplementary Information

Supplementary Information 1.

Supplementary Tables.

## Data Availability

The data is available at the following repository: https://figshare.com/s/a54eaaa299610353a2a3.
