# Author Correction: Longitudinal study of disease severity and external factors in cognitive failure after COVID-19 among Indonesian population

**DOI:** 10.1038/s41598-023-48244-9

**Published:** 2023-12-18

**Authors:** Bumi Herman, Martin Chi Sang Wong, Prawat Chantharit, Firdaus Fabrice Hannanu, Pramon Viwattanakulvanid

**Affiliations:** 1 College of Public Health Sciences, Chulalongkorn University, Bangkok, Thailand; 2 Department of Family and Preventive Medicine, Faculty of Medicine, Hasanuddin University, Makassar, Indonesia; 3 The Faculty of Medicine, JC School of Public Health, The Chinese University of Hongkong, Hong Kong, China; 4 The Faculty of Medicine, The Centre for Health Education and Health Promotion, The Chinese University of Hong Kong, Hong Kong, China; 5 School of Public Health, The Peking University, Beijing, China; 6 School of Public Health, Fudan University, Shanghai, China; 7 School of Public Health, The Chinese Academy of Medical Sciences and Peking Union Medical Colleges, Beijing, China; 8 Division of Infectious Diseases, Department of Medicine, Faculty of Medicine, Ramathibodi Hospital, Mahidol University, Bangkok, Thailand; 9 Department of Radiology, Brainstem Imaging Laboratory, Athinoula A. Martinos Center for Biomedical Imaging, Massachusetts General Hospital and Harvard Medical School, Boston, USA

Correction to: *Scientific Reports* https://doi.org/10.1038/s41598-023-46334-2, published online 08 November 2023

The original version of this Article contained an error in the spelling of the author Pramon Viwattanakulvanid which was incorrectly given as Pramon Viwattankulvanid.

The original Article has been corrected.

