# Supplementary material for: Longitudinal study of disease severity and external factors in cognitive failure after COVID-19 among Indonesian population
Source: Sci Rep. 2023 Nov 8;13:19405. doi: 10.1038/s41598-023-46334-2 (PMC10632387; doi:10.1038/s41598-023-46334-2)
Supplement: Supplementary file 1 — Supplementary Information 1. [file 41598_2023_46334_MOESM1_ESM.pdf]

Supplementary Data 1. Ordinal List of Categorical Variables
Other variables such as Age, Fatigue Severity Score (FSS), and Generalised Anxiety Disorder (GAD) Score were treated as continuous variables

Variable Values

Value		Label
Gender	0	Woman
	1	Man
Education	1	Up to Junior High School
	2	High School Graduate
	3	College/Diploma
	4	Undergraduate and Graduate
Employment	1.00	Unemployed
	2.00	Freelance
	3.00	Workers with certain working hours
	4.00	Medical Staff working at non-health facilities
	5.00	Medical Staff working at health facilities
Exercise	1.00	<1 time per week
	2.00	1-3 times per week
	3.00	>3 times per week
Smoking	1	Never Smoking
	2	Formerly Smoking
	3	Active Smoker
HT	0	No
	1	Yes, and Currently controlled by medication
	2	Yes, but remain uncontrolled
DM	0	No
	1	Yes, and Currently controlled by medication
	2	Yes, but remain uncontrolled
Loss of Smell	0	None
	1	up to 3 days
	2	4-7 days
	3	>7 days
Shortness of Breath	0	None
	1	up to 3 days
	2	4-7 days
	3	>7 days
Antivirus	1	<24 hours after diagnosis
	2	24-72 hours after diagnosis
	3	>72 hours after diagnosis

	4	Not Received
Days of Vaccination to Infection	1.00	> 30 Days After the Fourth Dose
	2.00	>60 days After the Fourth Dose
	3.00	>90 days After the Fourth Dose
	4.00	1-30 days after the Third Dose
	5.00	30-60 days after the Third Dose
	6.00	60-90 days after the Third Dose
	7.00	90-120 days after the Third Dose
	8.00	120-180 Days after the Third Dose
	9.00	>180 Days after the Third Dose
	10.00	1-30 days after the second dose
	11.00	30-60 days after the second dose
	12.00	60-90 days after the second dose
	13.00	90-120 days after the second dose
	14.00	120-180 days after the second dose
	15.00	>180 days after the second dose
	16.00	1-30 days after the first dose
	17.00	30-60 days after the first dose
	18.00	60-90 days after the first dose
	19.00	90-120 days after the first dose
	20.00	120-180 days after the first dose
	21.00	>180 days after the first dose
	22.00	Unvaccinated
The last dose received before the infection	1.00	After the fourth dose of the heterologous vaccine.'
	2.00	After the third dose of the heterologous vaccine
	3.00	After the third dose of the mRNA vaccine
	4.00	After the third dose of the viral vector vaccine
	5.00	after the third dose of inactivated vaccine
	6.00	after the second dose of the heterologous vaccine
	7.00	after the second dose of the mRNA vaccine
	8.00	after the second dose of the viral vector vaccine
	9.00	after the second dose of inactivated vaccine
	10.00	after the first dose of the mRNA vaccine
	11.00	after the first dose of the viral vector vaccine
	12.00	after the first dose of inactivated vaccine
	13	Unvaccinated
Previous Infection	1	Wild Type
	2	Alpha or Beta
	3	Delta
	4	Omicron

	5	No
The variant of the Latest Infection	1	Wild Type
	2	Alpha or Beta
	3	Delta
	4	Omicron
Unit of Treatment	1.00	Self-Isolation
	2.00	Isolation + Referred to Hospital
	3.00	Full hospitalisation
Body Mass Index Classification	1.00	Underweight
	2.00	Normal
	3.00	Overweight
	4.00	Obese I
	5.00	Obese II
CFQmean	.00	Below Mean
	1.00	Above Mean
