# Supplementary material for: Longitudinal study of disease severity and external factors in cognitive failure after COVID-19 among Indonesian population
Source: Sci Rep. 2023 Nov 8;13:19405. doi: 10.1038/s41598-023-46334-2 (PMC10632387; doi:10.1038/s41598-023-46334-2)

Supplementary Table S1. Model 1

Component based on bivariate selection
Severity =~ Shortness of Breath + Headache + Loss of Smell
Vaccination =~ dose_day + dose_type
comorbidity =~ DM + HT + Smoking + Exercise
demography =~ Employment + Education
Severity ~ Previous Infection + Age + Unit of Treatment + Vaccination + comorbidity + Latest Variant
Recent_Headache =~ Tension + Cluster + Migraine + VM + Vertigo + Iceprick
CFQ score ~ Severity + FSS + GAD + Recent_Headache + demography + comorbidity
'

Goodness Fit Indices
CFI 0.874
TLI 0.855
RMSEA 0.077
SRMR 0.088
MODEL DOES NOT FIT

Structure	Variables	Estimate	Standard Error of Estimate	z-value	p-value
**Latent Variables**					
Severity	Shortness of Breath duration	1			
	Loss of Smell duration	1.008	0.027	37.386	<0.001
	Headache duration	1.222	0.033	36.545	<0.001
Vaccination	Doses + Day to Infection	1			
	Doses + Type of Vaccine	0.648	0.011	60.385	<0.001
Demography	Employment	1			
	Higher Education	1.386	0.098	14.082	<0.001
Comorbidity	DM	1			
	HT	2.215	0.135	16.394	<0.001
	Smoking	0.701	0.043	16.304	<0.001
	Exercise	-0.206	0.029	-7.085	<0.001
Recent Headache	Tension Headache	1			
	Cluster Headache	1.113	0.012	91.233	<0.001
	Migraine	1.079	0.012	90.143	<0.001
	Vomit+ Nausea	0.936	0.012	81.022	<0.001
	Vertigo	1.102	0.012	89.835	<0.001
	Icepick Headache	1.093	0.012	91.833	<0.001
**Regression**					
Severity	No previous infection	-0.077	0.005	-14.889	<0.001
	Increase of Age	0.004	0.001	4.65	<0.001
	Unit of Treatment	0.15	0.024	6.266	<0.001
	Vaccination	0.005	0.002	2.979	0.003
	Comorbidity	-0.052	0.031	-1.651	0.099
	Latest Variant	-0.015	0.007	-2.073	0.038
CFQ score	More Severe Disease	0.591	0.589	1.004	0.315
	FSS score	0.214	0.031	6.833	<0.001
	GAD Score	1.465	0.087	16.805	<0.001
	Demography	-3.586	0.76	-4.718	<0.001
	Recent Headache	0.048	0.337	0.144	0.886
	Comorbidity	1.882	1.146	1.642	0.1
**Covariance**					
Vaccination	Comorbidity	-0.087	0.02	-4.4	<0.001
	Demography	-0.84	0.065	-12.994	<0.001
	Recent Headache	0.683	0.061	11.212	<0.001
Comorbidity	Demography	0.015	0.003	5.179	<0.001
	Recent Headache	0.03	0.004	7.503	<0.001
Demography	Recent Headache	-0.06	0.009	-6.845	<0.001

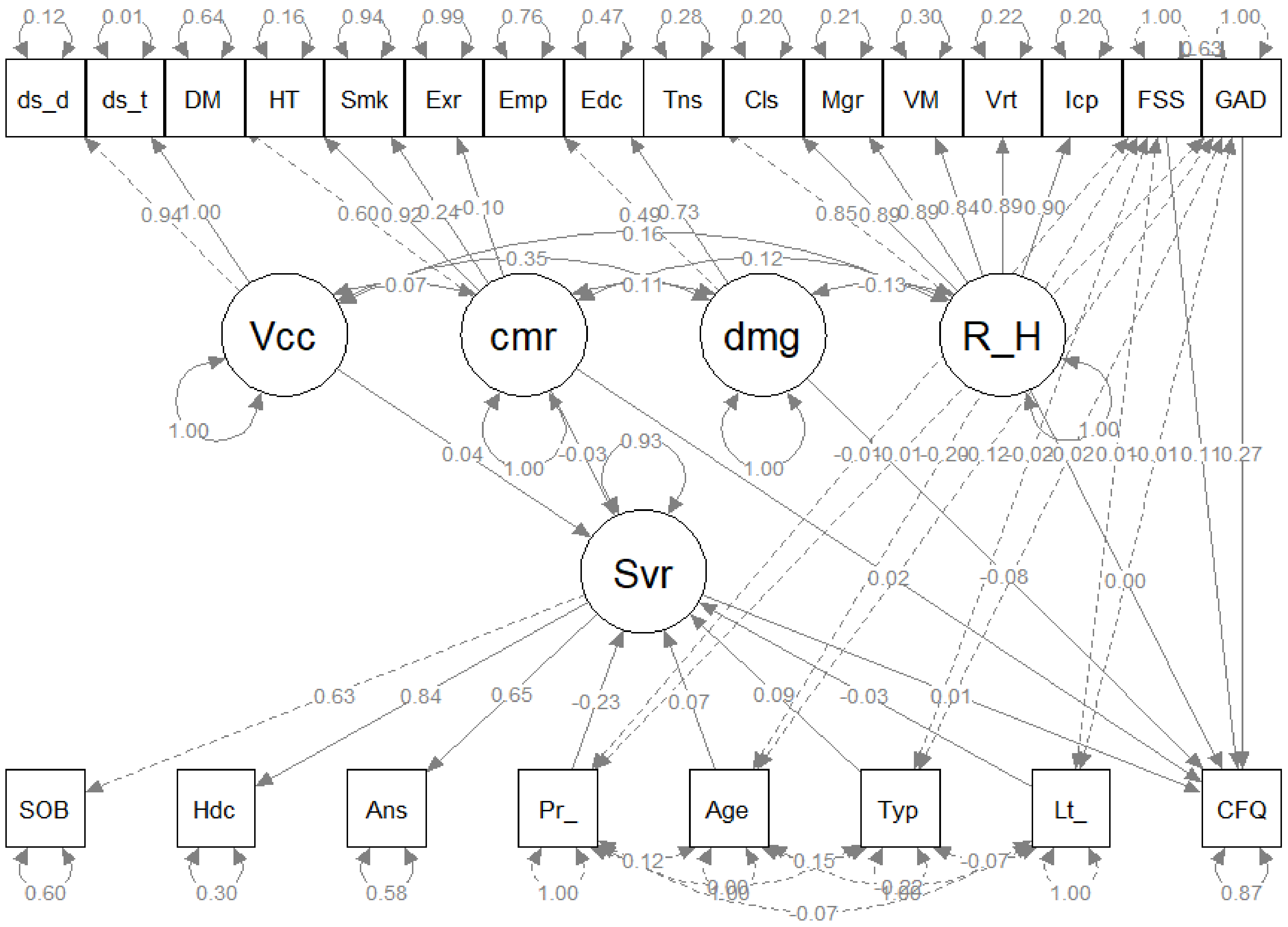

Supplementary Table S2. Model 2

Component based on bivariate selection
Severity =~ Shortness of Breath + Headache + Loss of Smell
Vaccination =~ dose_day + dose_type
demography =~ Employment + Education
Severity ~ Previous Infection + Age + Unit of Treatment + Vaccination+ Latest Variant
CFQ score ~ Severity + FSS + GAD + Recent_Headache + demography

Trimmed Variables
comorbidity =~ DM + HT + Smoking + Exercise
Recent_Headache =~ Tension + Cluster + Migraine + VM + Vertigo + Iceprick
'

Goodness Fit Indices
CFI 0.835
TLI 0.787
RMSEA 0.103
SRMR 0.096
MODEL DOES NOT FIT

Structure	Variables	Estimate	Standard Error of Estimate	z-value	p-value
**Latent Variables**					
Severity	Shortness of Breath duration	1			
	Loss of Smell duration	1.006	0.027	37.313	<0.001
	Headache duration	1.226	0.034	36.401	<0.001
Vaccination	Doses + Day to Infection	1			
	Doses + Type of Vaccine	0.658	0.012	52.883	<0.001
Demography	Employment	1			
	Higher Education	1.382	0.103	13.415	<0.001
**Regression**					
Severity	No previous infection	-0.076	0.005	-14.681	<0.001
	Increase of Age	0.004	0.001	4.449	0.008
	Unit of Treatment	0.149	0.024	6.228	<0.001
	Vaccination	0.005	0.002	3.223	0.001
	Latest Variant	-0.015	0.007	-1.996	0.046
CFQ score	More Severe Disease	0.664	0.589	1.128	0.26
	FSS score	0.216	0.031	6.901	<0.001
	GAD Score	1.461	0.087	16.76	<0.001
	Demography	-3.525	0.741	-4.758	<0.001
**Covariance**					
Vaccination	Demography	-0.829	0.066	-12.535	<0.001

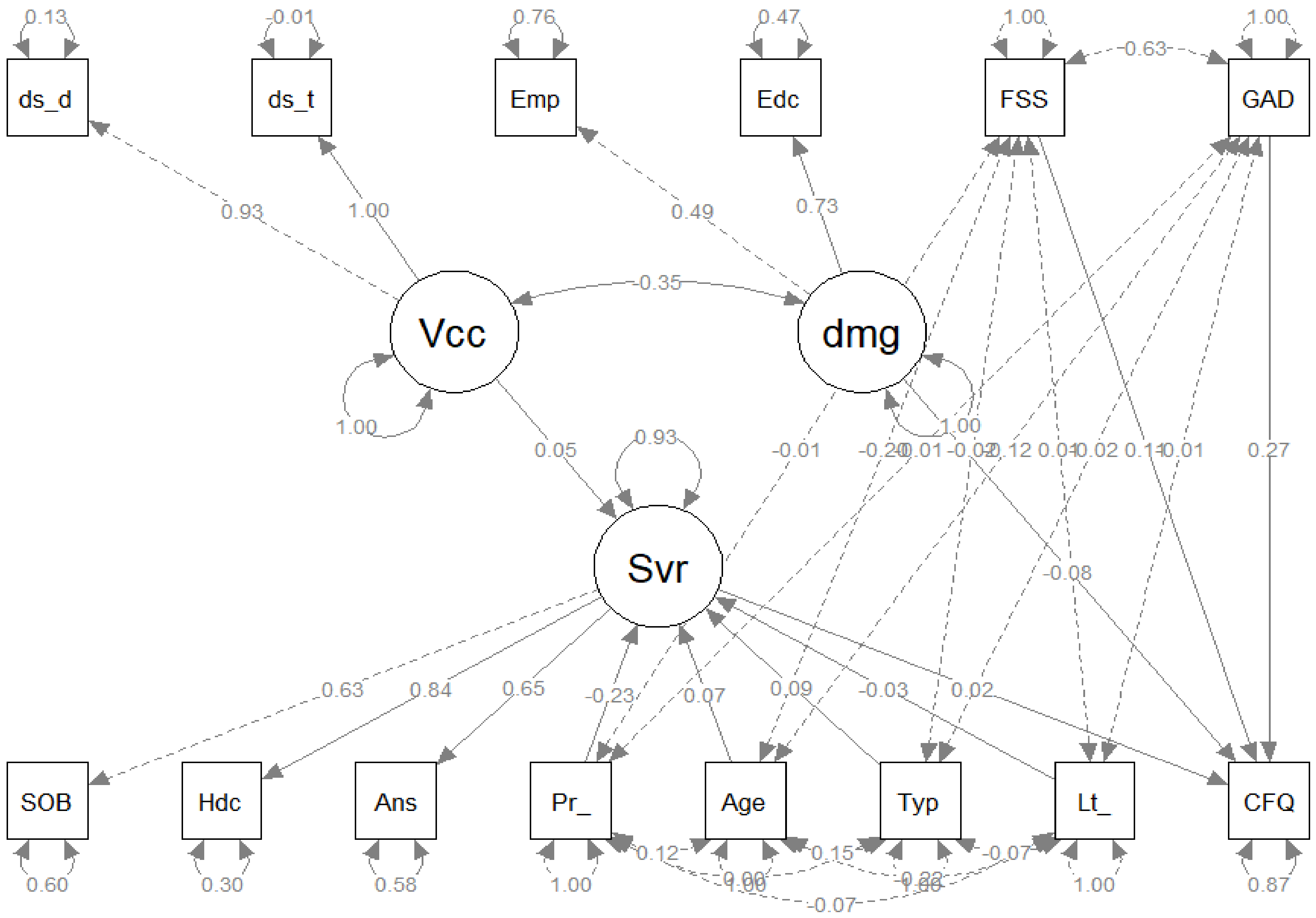

Supplementary Table S3. Model of Forgetfulness domain

Component

Severity =~ Shortness of Breath + Headache

Vaccination =~ dose_day + dose_type

demography =~ Employment + Education

Severity ~ Previous Infection + Age + Unit of Treatment + Vaccination

Forgetfulness~ Severity + FSS + GAD + Vaccination + demography + Age

Goodness Fit Indices

CFI 0.957

TLI 0.939

RMSEA 0.056

SRMR 0.044

MODEL ACCEPTABLE

Structure	Variables	Estimate	Standard Error of Estimate	z-value	p-value	Standardized estimate (β)	R-Square
**Latent Variables**							
Severity	Shortness of Breath duration	1				0.754	0.569
	Headache duration	0.87	0.06	14.567	<0.001	0.708	0.501
Vaccination	Doses + Day to Infection	1				0.946	0.895
	Doses + Type of Vaccine	0.637	0.011	58.924	<0.001	0.988	0.975
Demography	Employment	1				0.473	0.223
	Education	1.531	0.127	12.082	<0.001	0.768	0.59
**Regression**							
Severity	No previous infection	-0.076	0.007	-11.47	<0.001	-0.191	0.066
	Increase of Age	0.008	0.001	6.479	<0.001	0.104	
	Unit of Treatment	0.194	0.031	6.335	<0.001	0.101	
	Vaccination	0.014	0.002	6.31	<0.001	0.101	
Forgetfulness Score	More Severe Disease	0.125	0.219	0.57	0.569	0.009	0.108
	FSS score	0.116	0.013	8.918	<0.001	0.145	
	GAD Score	0.392	0.036	10.889	<0.001	0.175	
	Highly educated and working in a medical setting (Demography)	-0.071	0.336	-0.21	0.834	-0.004	
	Vaccination	0.212	0.027	7.769	<0.001	0.109	
	Age	-0.08	0.014	-5.818	<0.001	-0.075	
**Covariance**	Vaccination vs. Demography	-0.771	0.068	-11.38	<0.001	-0.331	

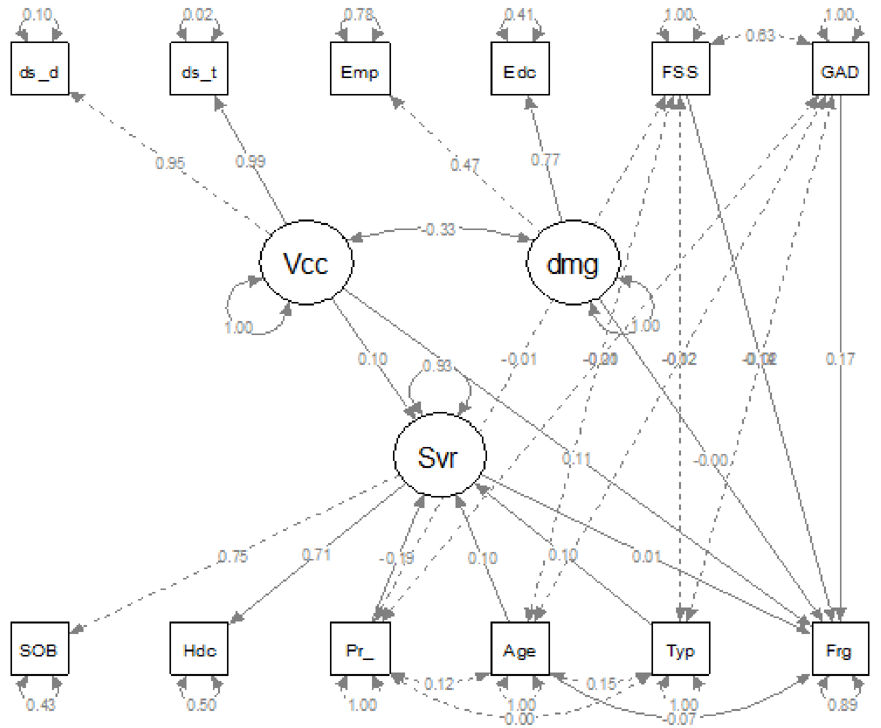

Supplementary Table S4. Model of Distraction domain

Component

Severity =~ Shortness of Breath + Headache

Vaccination =~ dose_day + dose_type

demography =~ Employment + Education

Severity ~ Previous Infection + Age + Unit of Treatment + Vaccination

Distraction~ Severity + FSS + GAD + Vaccination + demography + Age

Goodness Fit Indices

CFI 0.958

TLI 0.940

RMSEA 0.056

SRMR 0.044

MODEL ACCEPTABLE

Structure	Variables	Estimate	Standard Error of Estimate	z-value	p-value	Standardized estimate (β)	R-Square
**Latent Variables**							
Severity	Shortness of Breath duration	1				0.753	0.567
	Headache duration	0.873	0.06	14.581	<0.001	0.709	0.503
Vaccination	Doses + Day to Infection	1				0.94	0.883
	Doses + Type of Vaccine	0.646	0.011	59.542	<0.001	0.994	0.988
Demography	Employment	1				0.484	0.227
	Education	1.508	0.123	12.236	<0.001	0.73	0.581
**Regression**							
Severity	No previous infection	-0.076	0.007	-11.47	<0.001	-0.191	0.066
	Increase of Age	0.008	0.001	6.482	<0.001	0.104	
	Unit of Treatment	0.193	0.031	6.325	<0.001	0.101	
	Vaccination	0.014	0.002	6.333	<0.001	0.101	
Distraction Score	More Severe Disease	0.087	0.233	0.372	0.71	0.006	0.087
	FSS score	0.027	0.014	1.948	0.051	0.032	
	GAD Score	0.559	0.038	14.605	<0.001	0.237	
	Highly educated and working in a medical setting (Demography)	-0.274	0.357	-0.768	0.442	-0.013	
	Vaccination	0.255	0.029	8.738	<0.001	0.123	
	Age	-0.037	0.015	-2.514	0.012	-0.033	
**Covariance**	Vaccination vs. Demography	-0.773	0.067	-11.511	<0.001	-0.331	

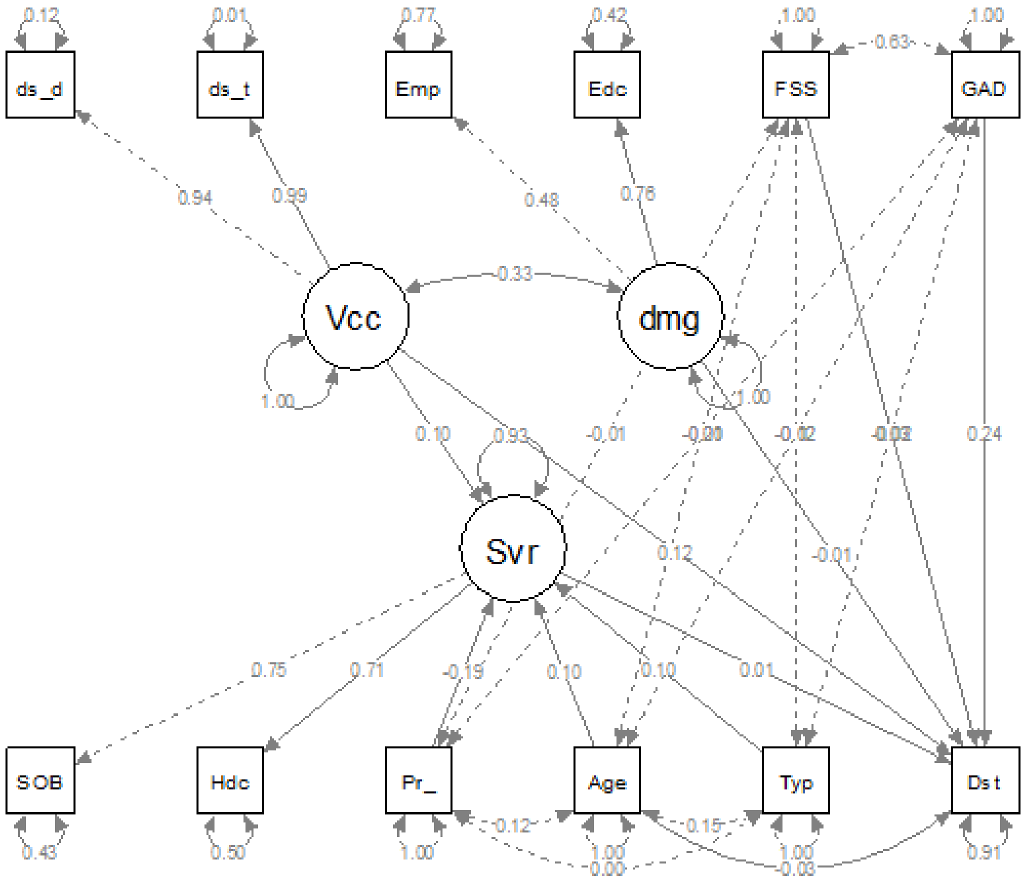

Supplementary Table S5. Model of False Trigger domain

Component

Severity =~ Shortness of Breath + Headache

Vaccination =~ dose_day + dose_type

demography =~ Employment + Education

Severity ~ Previous Infection + Age + Unit of Treatment + Vaccination

False Trigger~ Severity + FSS + GAD + Vaccination + demography + Age

Goodness Fit Indices

CFI 0.958

TLI 0.940

RMSEA 0.056

SRMR 0.044

MODEL ACCEPTABLE

Structure	Variables	Estimate	Standard Error of Estimate	z-value	p-value	Standardized estimate (β)	R-Square
**Latent Variables**							
Severity	Shortness of Breath duration	1				0.754	0.568
	Headache duration	0.871	0.06	14.583	<0.001	0.708	0.502
Vaccination	Doses + Day to Infection	1				0.941	0.885
	Doses + Type of Vaccine	0.645	0.011	60.004	<0.001	0.993	0.987
Demography	Employment	1				0.475	0.226
	Education	1.516	0.124	12.183	<0.001	0.764	0.584
**Regression**							
Severity	No previous infection	-0.076	0.007	-11.472	<0.001	-0.191	0.066
	Increase of Age	0.008	0.001	6.487	<0.001	0.104	
	Unit of Treatment	0.193	0.031	6.33	<0.001	0.101	
	Vaccination	0.014	0.002	6.336	<0.001	0.101	
False trigger Score	More Severe Disease	0.133	0.21	0.633	0.527	0.01	0.103
	FSS score	0.056	0.012	4.483	<0.001	0.073	
	GAD Score	0.499	0.034	14.497	<0.001	0.233	
	Highly educated and working in a medical setting (Demography)	-0.025	0.322	-0.078	0.937	-0.001	
	Vaccination	0.241	0.026	9.209	<0.001	0.129	
	Age	-0.04	0.013	-3.004	0.003	-0.039	
**Covariance**	Vaccination vs. Demography	-0.771	0.067	-11.464	<0.001	-0.331	

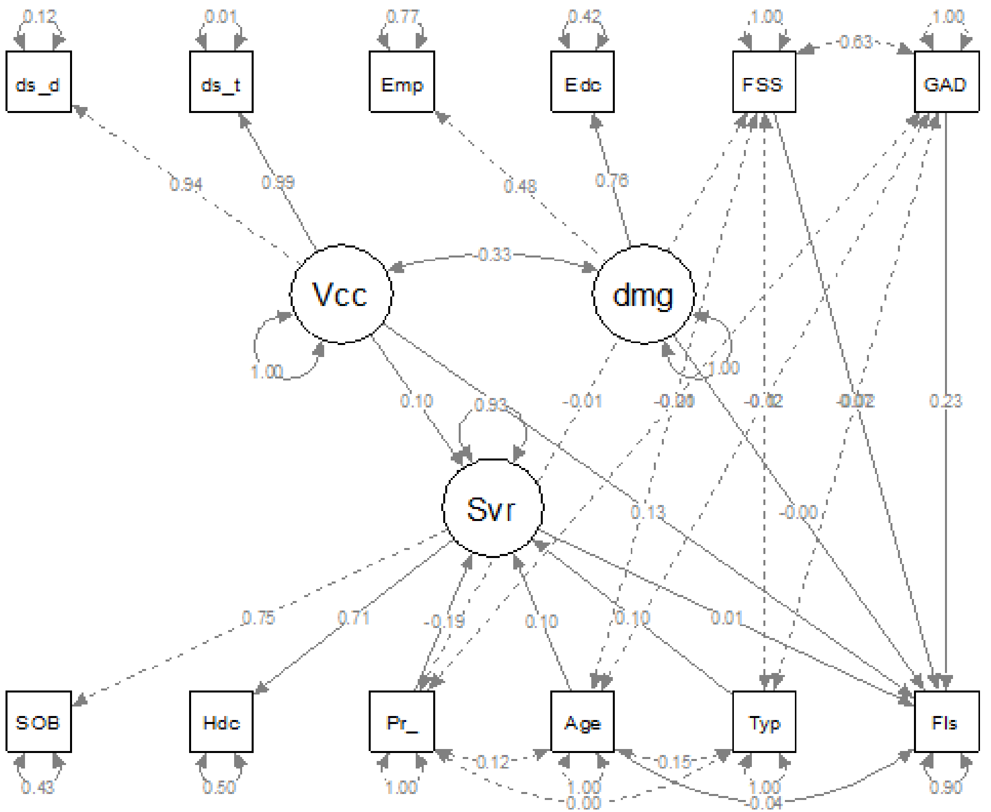

Supplementary Table S6.

6A. Gamma Ordinal Association between Variant of Latest Infection and Three Symptoms

Following the order, newer variance associated with lower duration of each symptoms

Loss of Smell

Variable		Latest Variant				Total	Value	p-value
		Wild Type	Alpha or Beta	Delta	Omicron			
Loss of Smell	None	96	25	147	470	738	-0.085	<0.001
	up tp 3 days	568	138	784	664	2154		
	4-7 days	475	178	719	796	2168		
	>7 days	73	18	366	163	620		
Total		1212	359	2016	2093	5680		

Shortness of Breath

Variable		Latest Variant				Total	Value	p-value
		Wild Type	Alpha or Beta	Delta	Omicron			
Shortness of Breath	None	189	39	440	821	1489	-0.171	<0.001
	up tp 3 days	736	192	800	769	2497		
	4-7 days	256	128	542	382	1308		
	>7 days	31	0	234	121	386		
Total		1212	359	2016	2093	5680		

Headache

Variable		Latest Variant				Total	Value	p-value
		Wild Type	Alpha or Beta	Delta	Omicron			
Headache	None	105	21	226	401	753	-0.092	<0.001
	up tp 3 days	701	156	978	1018	2853		
	4-7 days	383	175	520	524	1602		
	>7 days	23	7	292	150	472		
Total		1212	359	2016	2093	5680		

6B. Significance Level of each symptoms in association with CFQ Binary Class according to mean *tested by Gamma Ordinal Test*

Crosstab

		CFQmean			Value	p-value
		Below Mean	Above Mean	Total		
Loss of Smell	None	373	365	738	-0.006	**0.766**
	up tp 3 days	1064	1090	2154		
	4-7 days	1114	1054	2168		
	>7 days	302	318	620		
Total		2853	2827	5680		

Crosstab

		CFQmean			Value	p-value
		Below Mean	Above Mean	Total		
Shortness of Breath	None	796	693	1489	0.042	0.048
	up tp 3 days	1209	1288	2497		
	4-7 days	670	638	1308		
	>7 days	178	208	386		
Total		2853	2827	5680		

Crosstab

		CFQmean			Value	p-value
		Below Mean	Above Mean	Total		
Headache	None	397	356	753	0.045	0.041
	up tp 3 days	1442	1411	2853		
	4-7 days	797	805	1602		
	>7 days	217	255	472		
Total		2853	2827	5680		

Supplementary Table S7. Model 3

Component based on bivariate selection
Severity =~ Shortness of Breath + Headache + Loss of Smell
Vaccination =~ dose_day + dose_type
comorbidity =~ DM + HT + Smoking + Exercise
demography =~ Employment + Education
Severity ~ Previous Infection + Age + Unit of Treatment + Vaccination + comorbidity
Recent_Headache =~ Tension + Cluster + Migraine + VM + Vertigo + Iceprick
CFQ score ~ Severity + FSS + GAD + Recent_Headache + demography + comorbidity

Goodness Fit Indices
CFI 0.914
TLI 0.900
RMSEA 0.065
SRMR 0.073
MODEL ACCEPTABLE

Trimmed Variables from Model 1
Latest Variant

Structure	Variables	Estimate	Standard Error of Estimate	z-value	p-value
**Latent Variables**					
Severity	Shortness of Breath duration	1			
	Loss of Smell duration	1.006	0.027	37.401	<0.001
	Headache duration	1.224	0.033	36.553	<0.001
Vaccination	Doses + Day to Infection	1			
	Doses + Type of Vaccine	0.648	0.011	60.813	<0.001
Demography	Employment	1			
	Higher Education	1.386	0.098	14.083	<0.001
Comorbidity	DM	1			
	HT	2.215	0.135	16.393	<0.001
	Smoking	0.701	0.043	16.305	<0.001
	Exercise	-0.206	0.029	-7.084	<0.001
Recent Headache	Tension Headache	1			
	Cluster Headache	1.113	0.012	91.233	<0.001
	Migraine	1.079	0.012	90.143	<0.001
	Vomit+ Nausea	0.936	0.012	81.022	<0.001
	Vertigo	1.102	0.012	89.835	<0.001
	Icepick Headache	1.093	0.012	91.833	<0.001
**Regression**					
Severity	No previous infection	-0.077	0.005	-14.803	<0.001
	Increase of Age	0.005	0.001	4.953	<0.001
	Unit of Treatment	0.151	0.024	6.351	<0.001
	Vaccination	0.007	0.002	4.218	<0.001
	Comorbidity	-0.05	0.031	-1.601	0.109
CFQ score	More Severe Disease	0.581	0.588	0.988	0.323
	FSS score	0.214	0.031	6.833	<0.001
	GAD Score	1.465	0.087	16.805	<0.001
	Demography	-3.585	0.76	-4.715	<0.001
	Recent Headache	0.05	0.337	0.148	0.882
	Comorbidity	1.88	1.146	1.641	0.101
**Covariance**					
Vaccination	Comorbidity	-0.087	0.02	-4.4	<0.001
	Demography	-0.84	0.065	-12.999	<0.001
	Recent Headache	0.683	0.061	11.216	<0.001
Comorbidity	Demography	0.015	0.003	5.179	<0.001
	Recent Headache	0.03	0.004	7.504	<0.001
Demography	Recent Headache	-0.06	0.009	-6.846	<0.001

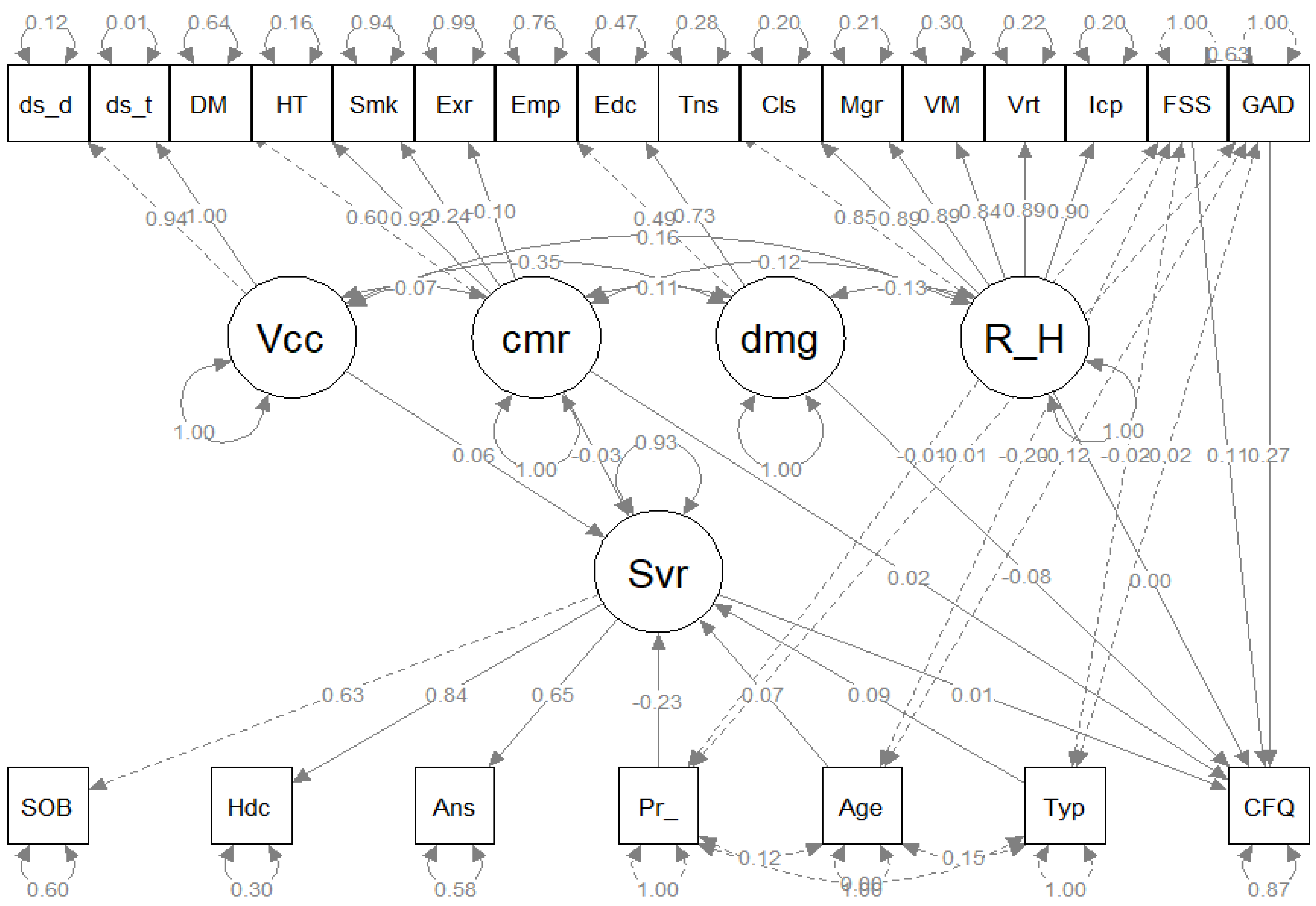

Supplememntary S8. Model 4

Component based on bivariate selection
Severity =~ Shortness of Breath + Headache
Vaccination =~ dose_day + dose_type
comorbidity =~ DM + HT + Smoking + Exercise
demography =~ Employment + Education
Severity ~ Previous Infection + Age + Unit of Treatment + Vaccination + comorbidity
Recent_Headache =~ Tension + Cluster + Migraine + VM + Vertigo + Iceprick
CFQ score ~ Severity + FSS + GAD + Recent_Headache + demography + comorbidity

Trimmed Variables from Model 3
Loss of Smell (as bivariate association to CFQ binary class showed p value 0.766)

Goodness Fit Indices
CFI 0.917
TLI 0.902
RMSEA 0.066
SRMR 0.071
MODEL ACCEPTABLE

Structure	Variables	Estimate	Standard Error of Estimate	z-value	p-value
**Latent Variables**					
Severity	Shortness of Breath duration	1			
	Headache duration	0.875	0.06	14.637	<0.001
Vaccination	Doses + Day to Infection	1			
	Doses + Type of Vaccine	0.647	0.01	62.043	<0.001
Demography	Employment	1			
	Higher Education	1.394	0.099	14.032	<0.001
Comorbidity	DM	1			
	HT	2.193	0.133	16.511	<0.001
	Smoking	0.702	0.043	16.33	<0.001
	Exercise	-0.205	0.029	-7.052	<0.001
Recent Headache	Tension Headache	1			
	Cluster Headache	1.113	0.012	91.235	<0.001
	Migraine	1.079	0.012	90.144	<0.001
	Vomit+ Nausea	0.936	0.012	81.023	<0.001
	Vertigo	1.102	0.012	89.836	<0.001
	Icepick Headache	1.093	0.012	91.834	<0.001
**Regression**					
Severity	No previous infection	-0.076	0.007	-11.472	<0.001
	Increase of Age	0.008	0.001	6.416	<0.001
	Unit of Treatment	0.193	0.031	6.312	<0.001
	Vaccination	0.014	0.002	6.423	<0.001
	Comorbidity	0.004	0.039	0.14	0.888
CFQ score	More Severe Disease	0.738	0.527	1.402	0.161
	FSS score	0.214	0.031	6.859	<0.001
	GAD Score	1.464	0.087	16.796	<0.001
	Demography	-3.532	0.762	-4.637	<0.001
	Recent Headache	0.022	0.337	0.064	0.949
	Comorbidity	1.841	1.145	1.608	0.108
**Covariance**					
Vaccination	Comorbidity	-0.088	0.02	-4.409	<0.001
	Demography	-0.837	0.065	-12.954	<0.001
	Recent Headache	0.684	0.061	11.231	<0.001
Comorbidity	Demography	0.015	0.003	5.262	<0.001
	Recent Headache	0.031	0.004	7.567	<0.001
Demography	Recent Headache	-0.059	0.009	-6.805	<0.001

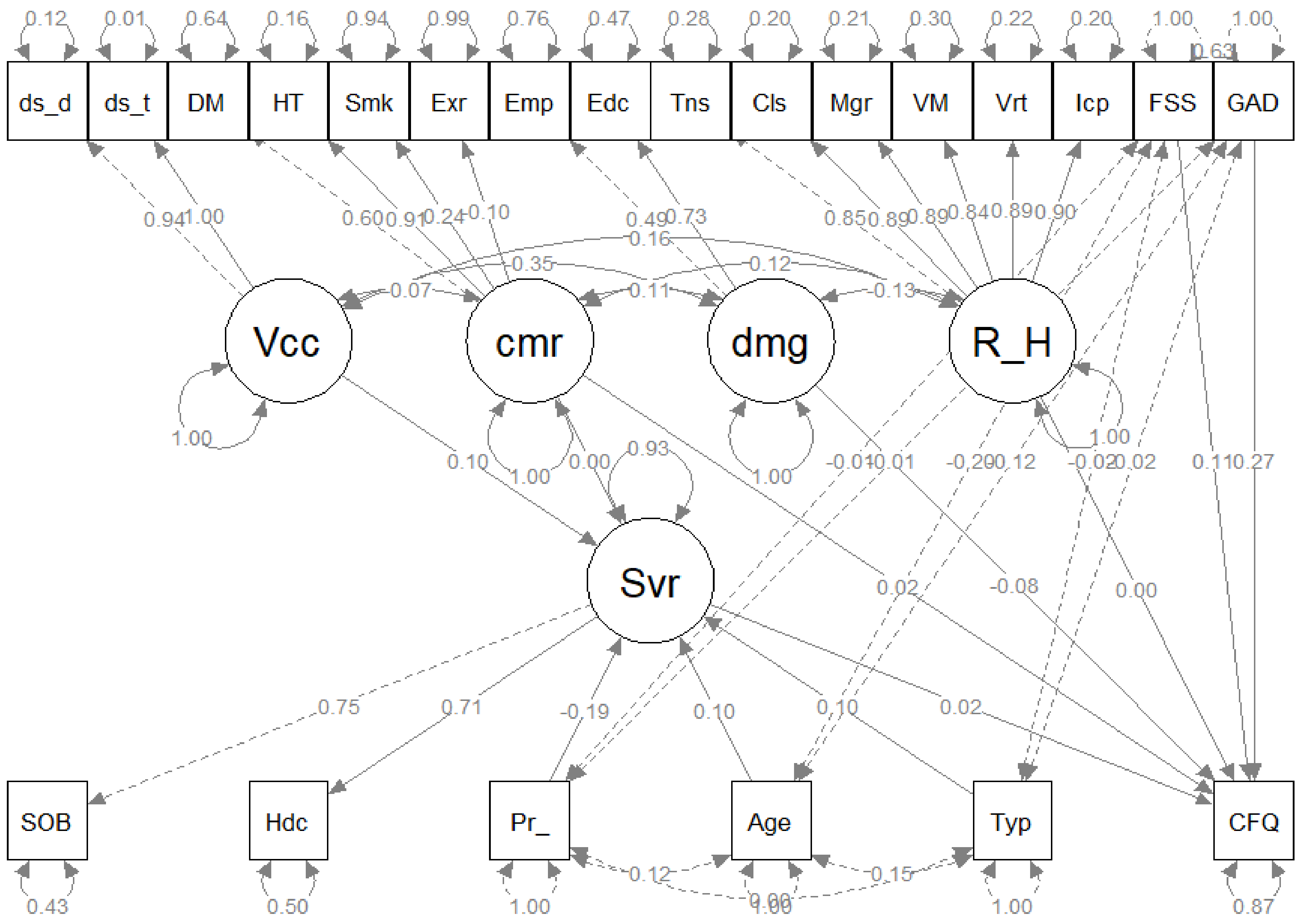

Supplementary Table S9
Association Between Antivirus Prescription and Unit of Treatment

p-value = <0.001

Antivirus (any type) * Type of Treatment Crosstabulation

			Type of Treatment			Total
			Home Isolation	Isolation + Referred to Hospital	Full hospitalization	
Antivirus [illegible]	<72 hours after diagnosis	Count	4266	514	46	4826
		% within Antivirus (any type)	88.4%	10.7%	0.9%	100.0%
		% within Type of Treatment	83.6%	97.2%	90.2%	85.9%
	>72 hours after diagnosis	Count	720	13	4	737
		% within Antivirus (any type)	97.7%	1.8%	.5%	100.0%
		% within Type of Treatment	14.1%	2.5%	7.8%	13.0%
	Not Received	Count	114	2	1	117
		% within Antivirus (any type)	97.4%	1.7%	.9%	100.0%
		% within Type of Treatment	2.2%	.4%	2.0%	2.1%

only 83.6% people who underwent home isolation received antivirus <72 hours after onset/diagnosis, and the proportion of people who did not receive antivirus was higher in self-isolation program

Supplementary Table S10. Additional Model of Vaccination to Recent Headache, and Cognitive Failure

Component

Vaccination =~ dose_day + dose_type
Recent_Headache =~ Tension + Cluster + Migraine + VM + Vertigo + Iceprick
Recent_Headache ~ Vaccination
CFQ Score~ Recent_Headache
CFQ Score ~ Vaccination
'

Structure	Variables	Estimate	Standard Error of Estimate	z-value	p-value
**Latent Variables**					
Vaccination	Doses + Day to Infection	1			
	Doses + Type of Vaccine	0.612	0.015	41.693	<0.001
Recent Headache	Tension Headache	1			
	Cluster Headache	1.113	0.012	91.197	<0.001
	Migraine	1.08	0.012	90.139	<0.001
	Vomit+ Nausea	0.936	0.012	80.931	<0.001
	Vertigo	1.102	0.012	89.717	<0.001
	Icepick Headache	1.093	0.012	91.786	<0.001
**Regression**					
Recent Headache	Vaccination	0.029	0.003	11.49	<0.001
CFQ score	Recent Headache	0.428	0.351	1.22	0.223
	Vaccination	0.657	0.064	10.293	<0.001
Indirect Effect	Vaccination to CFQ Via Recent Headache	0.281	0.228	1.235	0.217

Goodness Fit Indices
CFI 0.968
TLI 0.954
RMSEA 0.102
SRMR 0.031
MODEL ACCEPTABLE

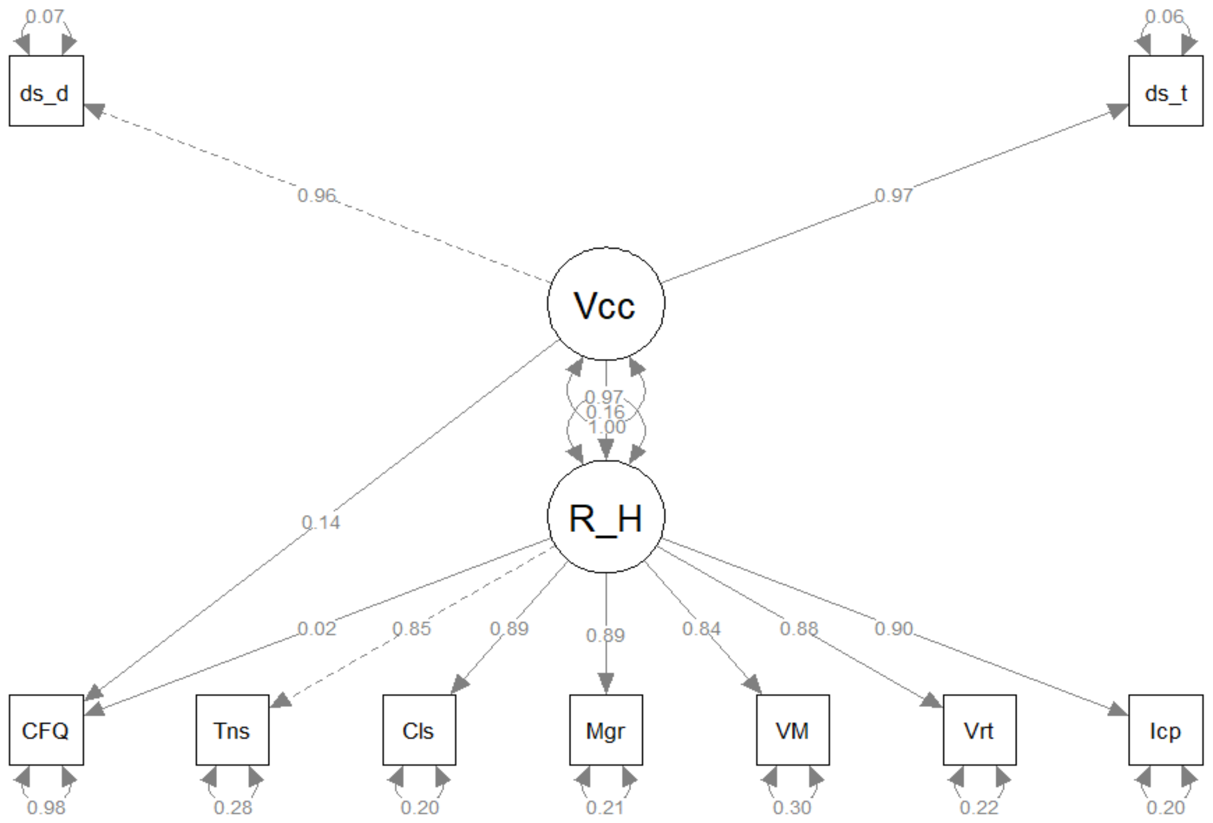

Supplement: Supplementary file 2 — Supplementary Tables. [file 41598_2023_46334_MOESM2_ESM.pdf]
